# Discontinuation vs. continuation of concomitant methotrexate in patients with rheumatoid arthritis on certolizumab pegol: results from a randomised, controlled trial

**DOI:** 10.1186/s13075-025-03548-1

**Published:** 2025-04-05

**Authors:** Shuji Asai, Toshihisa Kojima, Hajime Ishikawa, Nobumasa Miyake, Masanari Kodera, Hisanori Hasegawa, Yasumori Sobue, Yasuhide Kanayama, Hiromi Shimada, Yuji Hirano, Toshihiko Hidaka, Takayoshi Fujibayashi, Takuya Matsumoto, Tomonori Kobayakawa, Hidekata Yasuoka, Takefumi Kato, Masahiro Hanabayashi, Yuko Kaneko, Masahiro Tada, Koichi Murata, Kenta Misaki, Masahiko Ando, Yachiyo Kuwatsuka, Mochihito Suzuki, Kenya Terabe, Shiro Imagama

**Affiliations:** 1 Department of Orthopaedic Surgery and Rheumatology, Nagoya University Graduate School of Medicine, 65 Tsurumai-cho, Showa-ku, Nagoya, 466-8550 Aichi Japan; 2 Department of Orthopaedic Surgery and Rheumatology, National Hospital Organisation, Nagoya Medical Centre, Nagoya, Japan; 3 Department of Rheumatology, Niigata Rheumatic Centre, Shibata, Japan; 4 Miyake Orthopaedic Clinic, Shizuoka, Japan; 5 Department of Dermatology, Community Health Care Organisation, Chukyo Hospital, Nagoya, Japan; 6 Institute of Global Affairs, Institute of Science Tokyo, Tokyo, Japan; 7 Department of Orthopaedic Surgery, Japanese Red Cross Aichi Medical Centre Nagoya Daiichi Hospital, Nagoya, Japan; 8 Department of Orthopaedic Surgery and Rheumatology, Toyota Kosei Hospital, Toyota, Japan; 9 Division of Haematology, Rheumatology and Respiratory Medicine, Department of Internal Medicine, Faculty of Medicine, Kagawa University, Kagawa, Japan; 10 Department of Rheumatology, Toyohashi Municipal Hospital, Toyohashi, Japan; 11 Miyazaki-Zenjinkai Hospital, Miyazaki, Japan; 12 Department of Orthopaedic Surgery, Konan Kosei Hospital, Konan, Japan; 13 Department of Rheumatology, Shizuoka Kosei Hospital, Shizuoka, Japan; 14 Kobayakawa Orthopaedic and Rheumatologic Clinic, Fukuroi, Japan; 15 Division of Rheumatology, Department of Internal Medicine, Fujita Health University School of Medicine, Toyoake, Japan; 16 Kato Orthopaedic Clinic, Okazaki, Japan; 17 Department of Orthopaedic Surgery, Ichinomiya Municipal Hospital, Ichinomiya, Japan; 18 Division of Rheumatology, Department of Internal Medicine, Keio University School of Medicine, Tokyo, Japan; 19 Department of Orthopaedic Surgery, Osaka City General Hospital, Osaka, Japan; 20 Department of Advanced Medicine for Rheumatic Diseases, Kyoto University Graduate School of Medicine, Kyoto, Japan; 21 Department of Rheumatology, Kita-Harima Medical Centre, Ono, Japan; 22 Department of Advanced Medicine, Nagoya University Hospital, Nagoya, Japan

**Keywords:** Certolizumab pegol, Drug tapering, Methotrexate, Randomised controlled trial, Rheumatoid arthritis

## Abstract

**Objective:**

The present non-inferiority study was designed to compare the effect of discontinuing versus continuing methotrexate (MTX) alongside certolizumab pegol (CZP) on maintaining low disease activity (LDA) in rheumatoid arthritis (RA) patients already stable on combination therapy.

**Methods:**

This multicentre, open-label, randomised, controlled trial included RA patients with sustained LDA (Clinical Disease Activity Index [CDAI] ≤ 10) for ≥ 12 weeks with CZP + MTX. Patients were randomised 1:1 by computer to either continue MTX (CZP + MTX group) or discontinue MTX after a 12-week reduction period (CZP group) using a dynamic allocation strategy with the minimisation method. The primary endpoint was the proportion of patients maintaining LDA without a flare (i.e., a CDAI score > 10 or intervention with rescue treatments for any reason) at week 36 (24 weeks after MTX discontinuation). Non-inferiority is verified if the lower limit of the 90% confidence interval (CI) using normal approximation for the difference in the proportion of cases that maintained LDA at week 36 between the intervention group and control group exceeds the non-inferiority margin.

**Results:**

All 84 screened patients were randomised to the CZP + MTX group (*n* = 41) and CZP group (*n* = 43), and were included in the efficacy analysis. Proportions (90% CI) of patients who maintained LDA at week 36 were 85.4% (76.3 to 94.4%) in the CZP + MTX group and 83.7% (74.5 to 93.0%) in the CZP group. The difference (90% CI) between the two groups was − 1.6% (-14.6 to 11.3%), with the lower limit of the 90% CI exceeding the non-inferiority margin of -18%. Reported adverse events were broadly similar between the two groups. The proportion of patients with gastrointestinal symptoms, as assessed by a self-administered questionnaire, was significantly lower in the CZP group than in the CZP + MTX group at week 36 (2.4% vs. 15.8%, *P* = 0.034).

**Conclusion:**

Discontinuing concomitant MTX in RA patients on CZP is clinically feasible for maintaining LDA.

**Trial registration:**

Japan Registry of Clinical Trials (jRCTs041200048).

**Supplementary Information:**

The online version contains supplementary material available at 10.1186/s13075-025-03548-1.

## Introduction

The advent of novel therapeutic agents, including biologic/targeted synthetic disease-modifying antirheumatic drugs (b/tsDMARDs), alongside treatment strategies have enabled many rheumatoid arthritis (RA) patients to achieve their treatment targets, such as clinical remission or low disease activity (LDA) [1]. Combination therapy with b/tsDMARDs and methotrexate (MTX) has been shown to be highly effective in controlling disease activity in RA patients [2–6]. Given the chronic nature of RA treatment, the current challenge is to optimise cost-effectiveness and safety while maintaining treatment efficacy. Tapering bDMARDs has been shown to yield substantial cost savings without compromising disease control [7]. The TARA study, a randomised controlled trial (RCT), demonstrated that tapering either tumour necrosis factor (TNF) inhibitors or conventional synthetic DMARDs (csDMARDs), including MTX, first was similarly cost-effective. Notably, drug costs were significantly reduced in patients who tapered TNF inhibitors first, although this benefit was partially offset by increased overhead costs associated with reduced productivity [8].

Concomitant MTX can enhance the response to bDMARD therapy by inhibiting the clearance of bDMARDs and synergistic effects on the disease process itself [9]. However, the use of MTX may lead to adverse events (AEs), including gastrointestinal (GI) symptoms and liver dysfunction. Folic acid supplementation has been shown to mitigate these AEs [10]. We previously reported that, among Japanese RA patients receiving a median dose of 8 mg MTX, approximately 30% experienced GI symptoms, although roughly 90% of these patients were concurrently using folic acid [11, 12]. A recent study conducted in Japan found that the cumulative incidence of liver dysfunction in patients receiving MTX in combination with folic acid over a five-year period was 13% [13]. While discontinuation of concomitant MTX can reduce the above-mentioned AEs and increase safety in long-term treatment with bDMARDs, it may cause a relapse in disease activity. Previous studies, including RCTs, demonstrated that good disease activity can be maintained after discontinuing MTX in RA patients who achieved treatment targets with tocilizumab, an interleukin-6 inhibitor, and MTX [14–18]. On the other hand, there is little evidence for discontinuing concomitant MTX when used together with TNF inhibitors.

Certolizumab pegol (CZP) is a PEGylated Fab’ fragment of a humanised anti-human TNFα monoclonal antibody. In general, PEGylation decreases immunogenicity and lengthens the circulating half-life of antibodies due to decreased clearance [19]. CZP demonstrated clinical efficacy in monotherapy as well as with concomitant MTX in RA patients [20–23]. These findings open up the possibility of MTX discontinuation in RA patients if disease control can be maintained. To this end, the present non-inferiority study was designed to compare the effect of discontinuation and continuation of concomitant MTX on the maintenance of response in RA patients on CZP + MTX with sustained LDA.

## Patients and methods

### Study design

The present study, referred to as “the certolizumab Pegol treatment with Reducing and stoppIng MEthotrexate in patients with Rheumatoid Arthritis in stable LDA state” (hereafter, the PRIMERA study), is a multicentre, open-label, randomised, controlled 52-week trial conducted in Japan (Japan Registry of Clinical Trials identifier: jRCTs041200048; https://jrct.niph.go.jp/en-latest-detail/jRCTs041200048). This report analyses data from the first 36 weeks of the 52-week study, including the primary endpoint.

Patients were randomised 1:1 by computer to the MTX continuation (CZP + MTX) and MTX discontinuation (CZP) groups based on age, sex, disease duration, Clinical Disease Activity Index (CDAI), and MTX dose (< 10 or ≥ 10 mg/week) using a dynamic allocation strategy with the minimisation method. Study group assignment was performed by using a centralised, secure, and interactive web-based system (viedoc, Viedoc Technologies) accessible from each study site.

**Fig. 1:**
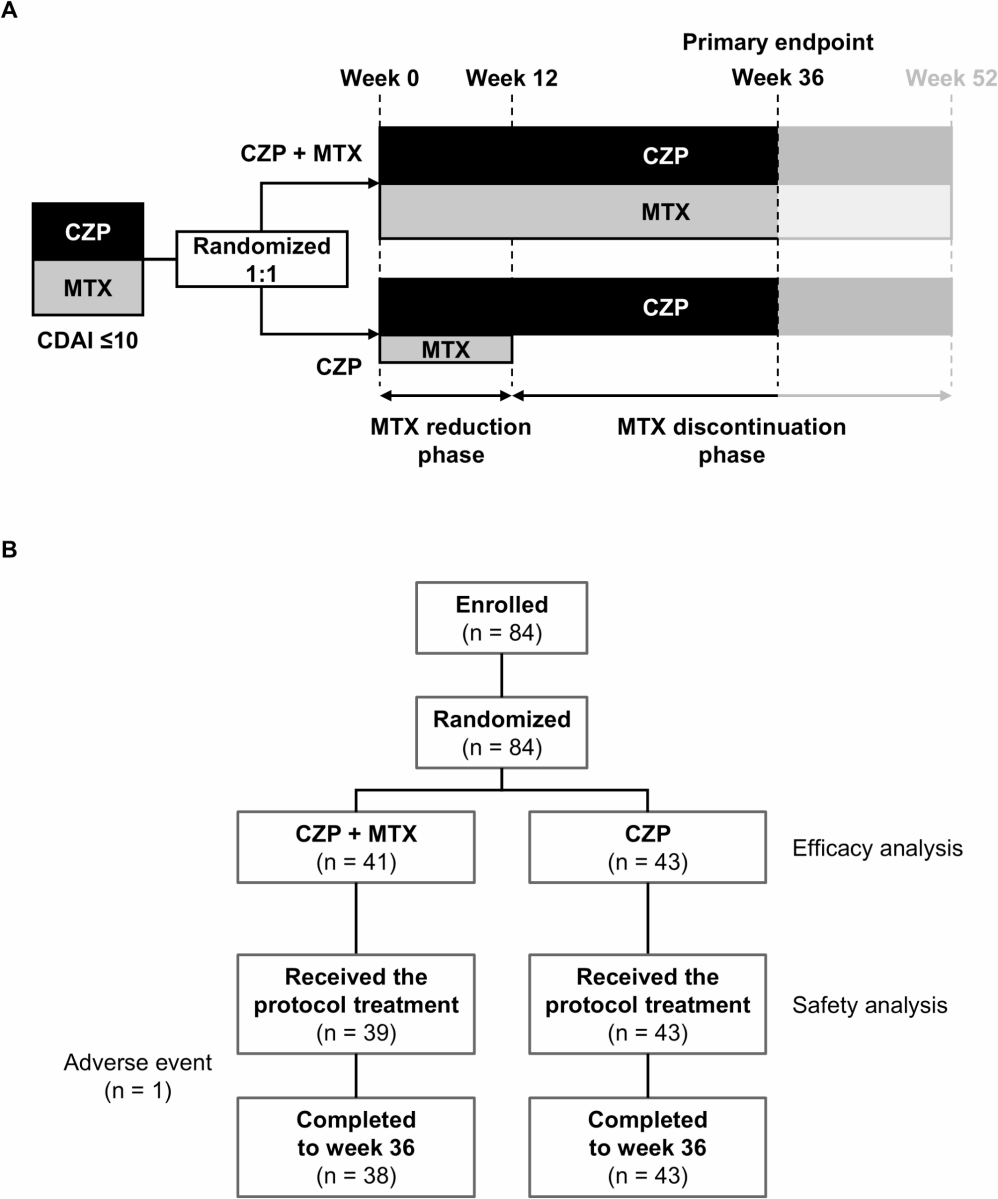
(**A**) Study design. (**B**) Patient disposition. CZP: certolizumab pegol; MTX: methotrexate

The study treatment scheme is shown in Fig. 1A. In the CZP group, the MTX dose was reduced by half at week 0, and discontinued at week 12 if LDA was maintained. Specifically, the MTX dose was halved and rounded up to the nearest multiple of 2 mg (e.g., 14 mg/week to 8 mg/week), since MTX orally administered to RA patients in Japan generally comes in 2 mg tablets. In both treatment groups, CZP and csDMARDs other than MTX were continued at a stable dose throughout the course of the study. Glucocorticoids were continued at a stable dose up to week 36 and allowed to taper after week 36. The use of oral analgesics (non-steroidal anti-inflammatory drugs, acetaminophen, pregabalin, and tramadol) was not prohibited during the study period. One or more of the following rescue treatments were performed if the CDAI score was > 10 or at the discretion of the investigator and/or upon patient request: increasing doses of or restarting MTX; increasing doses of or adding csDMARDs other than MTX or glucocorticoids; and administering an intra-articular injection of corticosteroids, hyaluronic acid, or lidocaine.

### Outcome measures

The primary endpoint was the proportion of patients maintaining LDA without a flare at week 36 (24 weeks after MTX discontinuation). Disease flare was defined as a CDAI score > 10 or intervention with rescue treatments for any reason. Secondary endpoints included the following parameters from week 0 to 52: disease activity (CDAI, Simple Disease Activity Index [SDAI], and Disease Activity Score with 28 joint counts with C-reactive protein [DAS28-CRP]), serum CRP and matrix metalloproteinase-3 (MMP-3) levels, physical function (Health Assessment Questionnaire Disability Index [HAQ-DI]), quality of life (EuroQol-5 dimension [EQ-5D]), and changes in modified total sharp score and atlantodental interval from week 0 to 52 as assessed by plain radiography (not shown in this report). Safety analysis included the incidence of AEs observed throughout the study period and GI symptoms (Frequency Scale for Symptoms of Gastroesophageal reflux disease [FSSG]) from week 0 to 52. The FSSG, a Japanese scale for gastroesophageal reflux disease (GERD) symptoms, is a self-administered questionnaire comprising 12 items rated on a five-point scale ranging from 0 (never) to 4 (always). The FSSG has been shown to correlate strongly with upper GI endoscopic findings. A cut-off score of 8 is used to diagnose GERD. Using locked data up to week 36, analyses related to the primary endpoint and corresponding analyses up to week 36 were performed. Analyses up to week 52 will be performed in the future using the final data.

### Patients

Participants were RA patients aged ≥ 20 years with sustained LDA (CDAI ≤ 10) for ≥ 12 weeks while receiving CZP + MTX. Patients met the 1987 American College of Rheumatology (ACR) classification criteria or the new ACR/European League Against Rheumatism (EULAR) diagnostic criteria for RA [24, 25]. Patients had to be receiving MTX orally at a stable dose of ≥ 6 mg/week (the minimum dose of MTX approved by the Ministry of Health, Labour and Welfare of Japan), and CZP at a stable dose according to the drug label in Japan, for ≥ 12 weeks prior to obtaining informed consent. Patients receiving csDMARDs other than MTX and/or glucocorticoids were eligible, but doses had to be stable for ≥ 12 weeks prior to obtaining informed consent.

The protocol was centrally reviewed and approved by the Certified Review Board of the Nagoya University Graduate School of Medicine (2020 − 0303), and was registered with the Japan Registry of Clinical Trials (jRCTs041200048). The present study was conducted in accordance with the Clinical Trials Act, and complied with the Declaration of Helsinki. Written informed consent was obtained from all patients.

### Statistical analyses

Assuming an expected success rate of 95% in the CZP + MTX group and 90% in the CZP group at week 36 with a non-inferiority margin of 18%, one-sided significance level of 0.05, and a power of 80%, the required number of cases was originally set at 102 (51 per group). Considering the possibility of some unevaluable cases, the target sample size was increased to 114 cases (57 per group). After the start of the study, the study protocol was amended because it was deemed difficult to reach the target sample size during the study period due in part to the coronavirus disease 2019 (COVID-19) pandemic. Accordingly, the power was set to 70%, and the required sample and target sample sizes were revised to 78 and 88 cases, respectively. Non-inferiority is verified if the lower limit of the 90% confidence interval (CI) using normal approximation for the difference in the proportion of cases that maintained LDA at week 36 between the intervention group and control group exceeds the non-inferiority margin. Other CIs for proportions were calculated using the Clopper–Pearson method.

For the analysis of secondary endpoints, proportions were compared using the χ^2^ test between the treatment groups. Estimated means and 95% CIs of repeatedly measured continuous items at each time point in both treatment groups were calculated using a linear mixed model with fixed effects of treatment group, time point, and interaction between treatment group and time point. To compare changes between treatment groups in each index at each time point, the Tukey–Kramer method was used. Statistical significance for secondary analyses was set at *P* < 0.05 (two-sided). Analyses were subjected to available case analysis, and missing data were not imputed. Analyses were conducted using Stata statistical software ver. 18 (Stata Corp LP, College Station, TX, USA) and SAS statistical software, V.9.4 (SAS Institute Corp, Cary, NC, USA).

## Results

### Patient disposition and baseline characteristics

Patient disposition is shown in Fig. 1B. Overall, 84 patients were enrolled at 20 institutions in Japan from January 1, 2021, to May 31, 2023. All 84 enrolled patients were randomised to the CZP + MTX group (*n* = 41) and the CZP group (*n* = 43), and were included in the efficacy analysis. Patient numbers were sufficient to allow analysis of the study objective (predetermined non-inferiority criteria were met). Of the 41 patients in the CZP + MTX group, one violated the protocol prior to week 0 and one was not seen after assignment. Thus, 39 patients in the CZP + MTX group and 41 patients in the CZP group started protocol treatment and were included in the safety analysis. Thirty-eight patients in the CZP + MTX group (one patient withdrew due to an AE) and all 43 patients in the CZP group completed to week 36. Table 1 shows the baseline (week 0) characteristics of patients included in the efficacy analyses. Demographic and clinical characteristics were balanced between the CZP + MTX and CZP groups.

**Table 1:** Patient characteristics at baseline (week 0)*

	Total (*n* = 84)	CZP + MTX (*n* = 41)	CZP (*n* = 43)
Age, years	59.2 ± 15.1	60.2 ± 14.2	58.2 ± 15.9
Female, no. (%)	70 (83.3)	35 (85.4)	35 (81.4)
Height, cm	158.2 ± 7.5	158.3 ± 6.8	158.2 ± 8.3
Weight, kg	54.6 ± 10.2	53.4 ± 8.5	55.7 ± 11.6
Disease duration, years	10.2 ± 7.6	11.7 ± 9.2	8.7 ± 5.4
RF positivity, no. (%)	83 (98.8)	40 (97.6)	43 (100)
ACPA positivity, no. (%)	63 (75.0)	28 (68.3)	35 (81.4)
Previous use of b/tsDMARDs, no. (%)	41 (48.8)	22 (53.7)	19 (44.2)
Duration of treatment with CZP, years	3.8 ± 2.1	3.4 ± 1.7	4.1 ± 2.4
MTX dose, mg/week	8.3 ± 2.4	7.9 ± 2.0	8.7 ± 2.6
Use of glucocorticoids, no. (%)	2 (2.4)	2 (4.9)	0 (0)
Use of csDMARDs other than MTX, no. (%)	20 (23.8)	7 (17.1)	13 (30.2)
28 TJC	0.4 ± 0.8	0.6 ± 1.0	0.3 ± 0.6
28 SJC	0.5 ± 1.0	0.7 ± 1.3	0.3 ± 0.7
PtGA, 0–100 mm scale	10.4 ± 14.1	10.6 ± 14.5	10.2 ± 13.9
PhGA, 0–100 mm scale	6.1 ± 6.2	5.8 ± 5.0	6.4 ± 7.2
CRP, mg/dL†	0.14 ± 0.57	0.07 ± 0.08	0.20 ± 0.79
MMP-3, ng/mL	48.8 ± 33.3	51.6 ± 45.3	46.2 ± 17.1
CDAI	2.6 ± 2.6	2.9 ± 2.9	2.3 ± 2.3
HAQ-DI	0.3 ± 0.5	0.3 ± 0.5	0.3 ± 0.5
EQ-5D	0.861 ± 0.149	0.862 ± 0.156	0.860 ± 0.145

*Except where indicated otherwise, values are presented as mean ± SD. †The upper limit of normal is 0.1 to 0.3 mg/dL. CZP: certolizumab pegol; MTX: methotrexate; RF: rheumatoid factor; ACPA: anti-citrullinated protein antibodies; b/tsDMARDs: biological/targeted-synthetic disease-modifying anti-rheumatic drugs; csDMARDs: conventional synthetic disease-modifying anti-rheumatic drugs; TJC: tender joint count; SJC: swollen joint count; PtGA: patient global assessment; PhGA: physician global assessment; CRP: C-reactive protein; MMP-3: matrix metalloproteinase-3; CDAI: Clinical Disease Activity Index; HAQ-DI: Health Assessment Questionnaire Disability Index; EQ-5D: EuroQol-5 dimension

### Efficacy

**Fig. 2:**
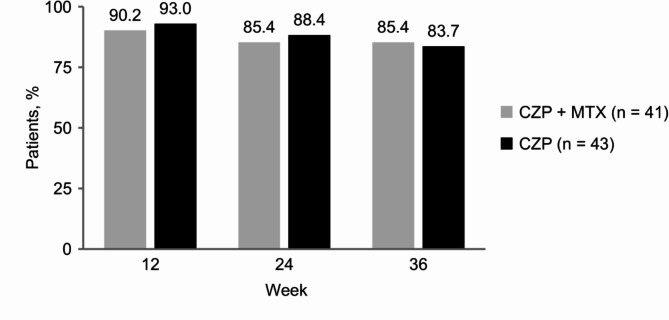
Proportion of patients maintaining low disease activity without a flare. Disease flare was defined as a clinical disease activity score > 10, intervention with rescue treatments, or dropout for any reason. CZP: certolizumab pegol; MTX: methotrexate

**Fig. 3:**
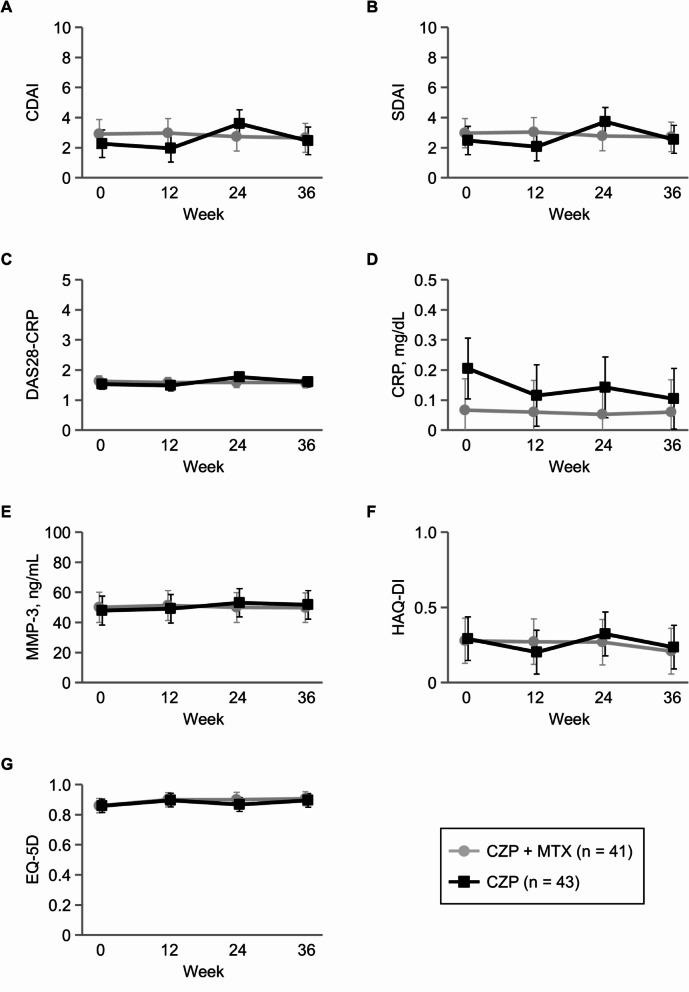
Estimated means and 95% confidence intervals for (**A**) Clinical Disease Activity Index (CDAI), (**B**) Simple Disease Activity Score (SDAI), (**C**) Disease Activity Score with 28 joint counts with C-reactive protein (DAS28-CRP), (**D**) CRP, (**E**) matrix metalloproteinase-3 (MMP-3), (**F**) Health Assessment Questionnaire Disability Index (HAQ-DI), and (**G**) EuroQol-5 dimension (EQ-5D). There was no significant difference for all comparisons between the two groups. CZP: certolizumab pegol; MTX: methotrexate

Proportions (90% CI) of patients who maintained LDA without a flare at week 36 were 85.4% (76.3 to 94.4%) in the CZP + MTX group and 83.7% (74.5 to 93.0%) in the CZP group (Fig. 2). The difference (90% CI) between the two groups was − 1.6% (-14.6 to 11.3%), with the lower limit of the 90% CI exceeding the non-inferiority margin of -18%. The present study met its primary endpoint by demonstrating non-inferiority of the CZP group compared with the CZP + MTX group. Comparison by the χ^2^ test showed no significant difference in the proportions (95% CI) of patients who maintained LDA without a flare between the CZP + MTX and CZP groups at week 12 (90.2% [76.9 to 97.3%] vs. 93.0% [80.9 to 98.5%], *p* = 0.645), week 24 (85.4% [70.8 to 94.4%] vs. 88.4% [74.9 to 96.1%], *p* = 0.683), and week 36 (85.4% [70.8 to 94.4%] vs. 83.7% [69.3 to 93.2%], *p* = 0.835). We also found no significant differences between the two groups in the estimated means and mean changes from baseline for CDAI, SDAI, DAS28-CRP, serum CRP and MMP-3 levels, HAQ-DI, and EQ-5D at all time points (Fig. 3 and Supplementary Material 1).

In the CZP group, a total of seven patients had a flare by week 36. Of these, two patients with CDAI scores > 10, and four patients who maintained CDAI scores ≤ 10, received rescue treatment, whereas one patient with a CDAI score > 10 requested not to (and did not) undergo rescue treatment. Both of the two patients with CDAI scores > 10 who received rescue treatment (one patient restarted MTX and received intra-articular injection, and another restarted MTX) regained LDA by week 36 (Supplementary Material 2).

### Safety

AEs reported during the period from week 0 to 36 are summarised in Table 2. AEs were broadly similar between the two groups, and no serious AEs were reported. Two cases of infection were reported in each group, and all four cases were COVID-19. One patient in the CZP + MTX group withdrew before week 24 due to haematuria. Safety analyses revealed no unexpected CZP safety issues.

**Table 2:** Safety*

	CZP + MTX (*n* = 39)	CZP (*n* = 43)
Total patients with ≥ 1 AE	7 (17.9)	5 (11.6)
AE	8 (19.5)	5 (11.6)
SAE	0 (0)	0 (0)
Gastrointestinal disorders	1 (2.4)	0 (0)
Infections and infestations	2 (4.9)	2 (4.7)
COVID-19	2 (4.9)	2 (4.7)
Injury, poisoning, and procedural complications	0 (0)	1 (2.3)
Investigations	1 (2.4)	0 (0)
Musculoskeletal and connective tissue disorders	2 (4.9)	0 (0)
Renal and urinary disorders	1 (2.4)	0 (0)
Respiratory, thoracic, and mediastinal disorders	1 (2.4)	0 (0)
Skin and subcutaneous tissue disorders	0 (0)	2 (4.7)

*Values are presented as number (%). CZP: certolizumab pegol; MTX: methotrexate; AE: adverse event; SAE: serious adverse event; COVID-19: coronavirus disease 2019

**Fig. 4:**
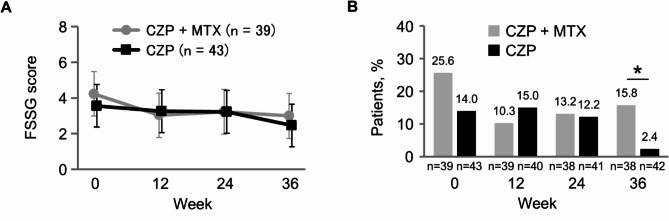
Gastrointestinal symptoms. (**A**) Estimated means and 95% confidence intervals for Frequency Scale for Symptoms of Gastroesophageal reflux disease (FSSG) score. (**B**) Proportion of patients with FSSG score ≥ 8. *P < 0.05 between the two groups. CZP: certolizumab pegol; MTX: methotrexate

There were no significant differences between the two groups in the estimated means and mean changes from baseline for FSSG score at all time points (Fig. 4A and Supplementary Material 3). The proportion of patients with FSSG score ≥ 8 was significantly lower in the CZP group than in the CZP + MTX group at week 36 (2.4% vs. 15.8%, *P* = 0.034), while there was no significant difference between the two groups at weeks 0, 12, and 24 (Fig. 4B and Supplementary Material 3).

## Discussion

The PRIMERA trial is an open-label RCT exploring the strategy for discontinuing concomitant MTX in RA patients treated with CZP + MTX. The results of the present study demonstrate that discontinuing MTX is non-inferior to continuing MTX in terms of maintenance of therapeutic effect in RA patients with sustained LDA treated with the combination therapy. The clinical feasibility of MTX discontinuation was supported by secondary efficacy analyses, which showed no significant differences in serum CRP and MMP-3 levels, HAQ-DI, or EQ-5D between the two groups throughout the study period.

Recent RCTs have examined the clinical feasibility of discontinuing concomitant csDMARDs, including MTX, in treatments with TNF inhibitors for RA patients. The SEAM-RA trial investigated whether sustained remission by combination therapy with etanercept and MTX can be maintained after discontinuing one or the other medication of the combination [26]. The efficacy of etanercept monotherapy (i.e., MTX discontinuation) was comparable to that of combination therapy in maintaining remission, although no statistical comparison was made between the two treatment groups. The CAMEO trial was designed to discontinue MTX regardless of disease activity after six months of combination therapy with etanercept and MTX [27]. Etanercept monotherapy (i.e., MTX discontinuation) was not non-inferior to etanercept + MTX with respect to change in DAS28 from six-month randomisation to 12 months. Subgroup analysis revealed that patients who achieved LDA at six months had a similar disease activity at 12 months, whether on monotherapy or combination therapy. Another RCT compared the effectiveness of CZP added to csDMARDs, including MTX, followed by continuing versus discontinuing background csDMARDs after achieving a treatment response [28]. CZP monotherapy (i.e., csDMARD discontinuation) was comparable to CZP + csDMARDs in terms of primary endpoints (change in DAS28 of ≥ 1.2 and/or DAS28 LDA achievement at 12 months after randomisation), but did not meet the non-inferiority criteria. The present study demonstrates for the first time the non-inferiority of MTX discontinuation versus MTX continuation in terms of maintenance of LDA in RA patients treated with a TNF inhibitor. Some of the differences relative to previous studies in patient background, study protocol, and primary endpoint may explain why the results of the present study showed non-inferiority.

Recent recommendations specify that patients be at treatment targets (remission in EULAR, and LDA or remission in ACR) for at least six months prior to tapering, although the optimal time at target prior to tapering has not been established [29, 30]. In the aforementioned RCT on CZP treatment, the protocol infers that patients sustained treatment targets for approximately 0–3 months before discontinuing MTX [28]. The present study included patients with sustained LDA for ≥ 12 weeks with CZP + MTX in clinical practice, but it was not designed to collect data on detailed durations of sustaining LDA at baseline. Given that patients were treated with CZP for 3.8 ± 2.1 years, it is likely that the patients had sustained LDA for an adequate time prior to enrolment in the study. Recent cohort studies suggest that a longer time at treatment targets prior to b/tsDMARD discontinuation (especially ≥ 6 months) predicts successful b/tsDMARD discontinuation [31, 32]. Further studies are needed to investigate the duration of stable disease control that can predict maintenance of good status after discontinuation of concomitant MTX.

Despite inclusion criteria that allowed patients to be on glucocorticoids, only two patients in the CZP + MTX group and none in the CZP group received glucocorticoids at baseline. Concomitant use of low-dose glucocorticoids has been shown to effectively increase remission rates in patients with early RA treated with csDMARDs [33, 34], and is recommended for short-term use only [29, 30]. Our previous observational studies have shown that remission achieved with concomitant glucocorticoids is less persistent than that achieved without concomitant glucocorticoids in RA patients treated with bDMARDs [35]. A recent cohort study showed that no glucocorticoid use at the time of bDMARD discontinuation is important for maintaining remission without using bDMARDs [31]. Sustaining LDA without concomitant glucocorticoids prior to MTX discontinuation may have led to a high proportion of patients maintaining LDA in the CZP group (83.7% at 24 weeks after discontinuing MTX) as well as the CZP + MTX group. Our findings align with the EULAR recommendation that glucocorticoids must be discontinued before considering tapering other DMARDs [30].

One of the most important concerns when considering discontinuation of a DMARD is whether the patient can quickly regain baseline status by rescue treatment (e.g., restarting a previous DMARD) if the disease flares up. Of the three patients with CDAI scores > 10 in the CZP group, two regained CDAI scores ≤ 10 upon restarting MTX, and one requested not to undergo rescue treatment. The same results were observed in our previous interventional study with a similar treatment protocol in patients treated with tocilizumab [17]. All three patients with CDAI scores > 10 after tapering MTX who received rescue treatment regained CDAI scores ≤ 10 by week 36. In the SEAM-RA trial on etanercept, of patients who restarted MTX because of disease-worsening after MTX discontinuation, 75% and 92% regained remission and LDA, respectively [26]. These results suggest that even if the disease flares up, the patient is likely to regain therapeutic targets by resuming MTX, which is reassuring for rheumatologists and patients who make the decision to discontinue MTX. It is important to monitor disease activity regularly to ensure that the timing of rescue treatment is not missed when discontinuing MTX in clinical practice.

The dose of MTX used in our study (mean ± SD, 8.3 ± 2.4 mg/week) was lower than that recommended for treating RA [36], even when adjusted for the typically lower body weight of Japanese patients compared to Western patients. The CONCERTO study conducted in Western countries showed an additive effect when MTX 10 mg/week was combined with a TNF inhibitor [37]. The MIRACLE study conducted in Asian countries showed that the efficacy of a TNF inhibitor combined with a low methotrexate dose (6 to 8 mg/week) was not inferior to that with the maximum tolerated methotrexate dose [38]. These findings suggest that concomitant MTX at a dose as low as 8 mg/week may be sufficient for Japanese patients treated with TNF inhibitors.

There were no unexpected safety signals reported in the present study, and no new safety signals were identified. There was no clear difference in the incidence of AEs between the CZP + MTX and CZP groups, probably due to the limited observation period in patients who had tolerated MTX for a long time before enrolment. Interestingly, the proportion of patients with GI symptoms, as assessed by a self-administered questionnaire, was significantly lower in the CZP group than in the CZP + MTX group at week 36. Our previous interventional study on tocilizumab also showed that tapering MTX resulted in a decreased prevalence of GI symptoms [17]. GI symptoms have been reported to decrease the quality of life of RA patients [39], and improvement of GI symptoms is important to improve quality of life during RA treatment. Although data from week 52 of the PRIMERA study need to be analysed, discontinuation of concomitant MTX may be beneficial in terms of reducing GI symptoms in patients treated with CZP.

The present study has some noteworthy limitations. First, it was conducted as an open-label study where both patients and evaluators were aware of the reduction and discontinuation of MTX, potentially influencing the assessments. The definition of disease flare included rescue treatment interventions upon patient request, aiming to mitigate investigator bias. Indeed, four of the seven patients in the CZP group who had a flare by week 36 received rescue treatment at the investigator’s discretion or upon patient request, even though they had maintained a CDAI score of ≤ 10. We believe our assessment approach likely reflects more closely what occurs in clinical practice. Second, potential selection biases are present. The present study included patients who maintained LDA in clinical practice. It is possible that patients suitable for MTX discontinuation were selected, which may have resulted in favourable outcomes. Further studies are needed to investigate on what basis rheumatologists decide to discontinue MTX. Third, the observation period was restricted despite the chronic nature of patient conditions requiring long-term treatment. Previous RCTs examining MTX discontinuation in bDMARDs therapy have set their primary endpoints between 12 and 56 weeks (with most at 12 to 24 weeks) after MTX discontinuation [14–16, 26–28]. Therefore, we considered the duration of 36 weeks (i.e., 24 weeks after MTX discontinuation) to be sufficient for assessing the effects of MTX discontinuation as the primary outcome. Finally, due to the small sample size for secondary analyses, the significance of certain findings may change with a larger dataset.

## Conclusions

The results of the present study demonstrate that discontinuing MTX is non-inferior to continuing MTX in terms of subsequent maintenance of LDA sustained with CZP + MTX therapy, and discontinuing concomitant MTX is clinically feasible for RA patients treated with CZP.

## Electronic supplementary material

Below is the link to the electronic supplementary material.

Supplementary Material 1

## Data Availability

The data that support the findings of this study are available from the corresponding author upon reasonable request.

## Abbreviations

MTX: Methotrexate
b/tsDMARDs: Biologic/targeted synthetic disease-modifying antirheumatic drugs
RA: Rheumatoid arthritis
LDA: Low disease activity
GI: Gastrointestinal
SEAs: Serious adverse events
AEs: Adverse events
RCTs: Randomised controlled trials
TNF: Tumour necrosis factor
CZP: Certolizumab pegol
CDAI: Clinical Disease Activity Index
csDMARDs: Conventional synthetic disease-modifying antirheumatic drugs
SDAI: Simple Disease Activity Index
DAS28-CRP: Disease Activity Score with 28 joint counts with C-reactive protein
MMP-3: Matrix metalloproteinase-3
HAQ-DI: Health Assessment Questionnaire Disability Index
EQ-5D: EuroQol-5 dimension
FSSG: Frequency Scale for Symptoms of Gastroesophageal reflux disease
GERD: Gastroesophageal reflux disease
ACR: American College of Rheumatology
EULAR: European League Against Rheumatism
COVID-19: Coronavirus disease 2019
CI: Confidence interval

## References

[1] Smolen JS, Breedveld FC, Burmester GR, Bykerk V, Dougados M, Emery P, et al. Treating rheumatoid arthritis to target: 2014 update of the recommendations of an international task force. Ann Rheum Dis. 2016;75(1):3–15. 25969430 10.1136/annrheumdis-2015-207524 PMC4717393

[2] Klareskog L, van der Heijde D, de Jager JP, Gough A, Kalden J, Malaise M, et al. Therapeutic effect of the combination of etanercept and methotrexate compared with each treatment alone in patients with rheumatoid arthritis: double-blind randomised controlled trial. Lancet. 2004;363(9410):675–81. 15001324 10.1016/S0140-6736(04)15640-7

[3] Breedveld FC, Weisman MH, Kavanaugh AF, Cohen SB, Pavelka K, van Vollenhoven R, et al. The PREMIER study: A multicenter, randomized, double-blind clinical trial of combination therapy with adalimumab plus methotrexate versus methotrexate alone or adalimumab alone in patients with early, aggressive rheumatoid arthritis who had not had previous methotrexate treatment. Arthritis Rheum. 2006;54(1):26–37. 16385520 10.1002/art.21519

[4] Kaneko Y, Atsumi T, Tanaka Y, Inoo M, Kobayashi-Haraoka H, Amano K, et al. Comparison of adding Tocilizumab to methotrexate with switching to Tocilizumab in patients with rheumatoid arthritis with inadequate response to methotrexate: 52-week results from a prospective, randomised, controlled study (SURPRISE study). Ann Rheum Dis. 2016;75(11):1917–23. 26733110 10.1136/annrheumdis-2015-208426 PMC5099201

[5] Kremer JM, Genant HK, Moreland LW, Russell AS, Emery P, Abud-Mendoza C, et al. Results of a two-year followup study of patients with rheumatoid arthritis who received a combination of abatacept and methotrexate. Arthritis Rheum. 2008;58(4):953–63. 18383390 10.1002/art.23397

[6] Taylor PC, Keystone EC, van der Heijde D, Weinblatt ME, Del Carmen Morales L, Reyes Gonzaga J, et al. Baricitinib versus placebo or adalimumab in rheumatoid arthritis. N Engl J Med. 2017;376(7):652–62. 28199814 10.1056/NEJMoa1608345

[7] van Esveld L, Cox JM, Kuijper TM, Bosch TM, Weel-Koenders AE. Cost-utility analysis of tapering strategies of biologicals in rheumatoid arthritis patients in the Netherlands. Ann Rheum Dis. 2023;82(10):1296–306. 37423648 10.1136/ard-2023-224190

[8] van Mulligen E, Weel AE, Kuijper TM, Denissen NHAM, Gerards AH, de Jager MH, et al. Two-year cost effectiveness between two gradual tapering strategies in rheumatoid arthritis: cost-utility analysis of the TARA trial. Ann Rheum Dis. 2020;79(12):1550–6. 32907801 10.1136/annrheumdis-2020-217528 PMC7677489

[9] Ruderman EM. The role of concomitant methotrexate in biologic therapy for rheumatoid arthritis. Bull Hosp Jt Dis (2013). 2013;71(Suppl 1):S29–32. 24219038

[10] Singh JA. Folic acid supplementation for rheumatoid arthritis patients on methotrexate: the good gets better. Cochrane Database Syst Rev. 2013; 2013(7):ED000063. 10.1002/14651858.ED000063 PMC10846356 24151647

[11] Asai S, Nagai K, Takahashi N, Watanabe T, Matsumoto T, Asai N, et al. Influence of methotrexate on Gastrointestinal symptoms in patients with rheumatoid arthritis. Int J Rheum Dis. 2019;22(2):207–13. 30168274 10.1111/1756-185X.13380

[12] Asai S, Takahashi N, Nagai K, Watanabe T, Matsumoto T, Asai N, et al. Influence of Gastrointestinal symptoms on patient global assessment in patients with rheumatoid arthritis. SN Compr Clin Med. 2020;2(5):619–26.

[13] Mori S, Arima N, Ito M, Ueki Y, Abe Y, Aoyagi K, et al. Incidence, predictive factors and severity of methotrexate-related liver injury in rheumatoid arthritis: a longitudinal cohort study. Rheumatol Adv Pract. 2020;4(2):rkaa020. 33134809 10.1093/rap/rkaa020 PMC7585403

[14] Kremer JM, Rigby W, Singer NG, Birchwood C, Gill D, Reiss W, et al. Sustained response following discontinuation of methotrexate in patients with rheumatoid arthritis treated with subcutaneous Tocilizumab: results from a randomized, controlled trial. Arthritis Rheumatol. 2018;70(8):1200–8. 29575803 10.1002/art.40493

[15] Edwards CJ, Östör AJK, Naisbett-Groet B, Kiely P. Tapering versus steady-state methotrexate in combination with Tocilizumab for rheumatoid arthritis: a randomized, double-blind trial. Rheumatology (Oxford). 2018;57(1):84–91. 29155973 10.1093/rheumatology/kex358

[16] Pablos JL, Navarro F, Blanco FJ, Román-Ivorra JA, Alonso A, Martín Mola E, et al. Efficacy of Tocilizumab monotherapy after response to combined Tocilizumab and methotrexate in patients with rheumatoid arthritis: the randomised JUST-ACT study. Clin Exp Rheumatol. 2019;37(3):437–44. 30299241

[17] Asai S, Hayashi M, Hanabayashi M, Kanayama Y, Takemoto T, Yabe Y, et al. Discontinuation of concomitant methotrexate in Japanese patients with rheumatoid arthritis treated with Tocilizumab: an interventional study. Mod Rheumatol. 2020;30:434–41. 31390271 10.1080/14397595.2019.1641934

[18] Asai S, Takahashi N, Hayashi M, Hanabayashi M, Kanayama Y, Takemoto T, et al. Predictors of disease flare after discontinuation of concomitant methotrexate in Japanese patients with rheumatoid arthritis treated with Tocilizumab. Joint Bone Spine. 2020;87(6):596–602. 32534200 10.1016/j.jbspin.2020.06.001

[19] Chapman AP. PEGylated antibodies and antibody fragments for improved therapy: a review. Adv Drug Deliv Rev. 2002;54(4):531–45. 12052713 10.1016/s0169-409x(02)00026-1

[20] Weinblatt ME, Fleischmann R, Huizinga TW, Emery P, Pope J, Massarotti EM, et al. Efficacy and safety of certolizumab Pegol in a broad population of patients with active rheumatoid arthritis: results from the REALISTIC phase IIIb study. Rheumatology (Oxford). 2012;51(12):2204–14. 22923753 10.1093/rheumatology/kes150

[21] Yamamoto K, Takeuchi T, Yamanaka H, Ishiguro N, Tanaka Y, Eguchi K, et al. Efficacy and safety of certolizumab Pegol plus methotrexate in Japanese rheumatoid arthritis patients with an inadequate response to methotrexate: the J-RAPID randomized, placebo-controlled trial. Mod Rheumatol. 2014;24(5):715–24. 24313916 10.3109/14397595.2013.864224

[22] Yamamoto K, Takeuchi T, Yamanaka H, Ishiguro N, Tanaka Y, Eguchi K, et al. Efficacy and safety of certolizumab Pegol without methotrexate co-administration in Japanese patients with active rheumatoid arthritis: the HIKARI randomized, placebo-controlled trial. Mod Rheumatol. 2014;24(4):552–60. 24981319 10.3109/14397595.2013.843764

[23] Fleischmann R, van Vollenhoven RF, Vencovský J, Alten R, Davies O, Mountian I, et al. Long-Term maintenance of certolizumab Pegol safety and efficacy, in combination with methotrexate and as monotherapy, in rheumatoid arthritis patients. Rheumatol Ther. 2017;4(1):57–69. 28353191 10.1007/s40744-017-0060-8 PMC5443729

[24] Arnett FC, Edworthy SM, Bloch DA, McShane DJ, Fries JF, Cooper NS, et al. The American rheumatism association 1987 revised criteria for the classification of rheumatoid arthritis. Arthritis Rheum. 1988;31(3):315–24. 3358796 10.1002/art.1780310302

[25] Aletaha D, Neogi T, Silman AJ, Funovits J, Felson DT, Bingham CO, et al. 2010 Rheumatoid arthritis classification criteria: an American college of rheumatology/european league against rheumatism collaborative initiative. Arthritis Rheum. 2010;62(9):2569–81. 20872595 10.1002/art.27584

[26] Curtis JR, Emery P, Karis E, Haraoui B, Bykerk V, Yen PK, et al. Etanercept or methotrexate withdrawal in rheumatoid arthritis patients in sustained remission. Arthritis Rheumatol. 2021;73(5):759–68. 33205906 10.1002/art.41589 PMC8251940

[27] Pope JE, Haraoui B, Thorne JC, Vieira A, Poulin-Costello M, Keystone EC. The Canadian methotrexate and etanercept outcome study: a randomised trial of discontinuing versus continuing methotrexate after 6 months of etanercept and methotrexate therapy in rheumatoid arthritis. Ann Rheum Dis. 2014;73(12):2144–51. 23979914 10.1136/annrheumdis-2013-203684 PMC4251190

[28] Pope J, Rampakakis E, Vaillancourt J, Bessette L, Lazovskis J, Haraoui B, et al. An open-label randomized controlled trial of DMARD withdrawal in RA patients achieving therapeutic response with certolizumab Pegol combined with DMARDs. Rheumatology (Oxford). 2020;59(7):1522–8. 31628486 10.1093/rheumatology/kez470

[29] Fraenkel L, Bathon JM, England BR, St Clair EW, Arayssi T, Carandang K, et al. 2021 American college of rheumatology guideline for the treatment of rheumatoid arthritis. Arthritis Rheumatol. 2021;73(7):1108–23. 34101376 10.1002/art.41752

[30] Smolen JS, Landewé RBM, Bergstra SA, Kerschbaumer A, Sepriano A, Aletaha D, et al. EULAR recommendations for the management of rheumatoid arthritis with synthetic and biological disease-modifying antirheumatic drugs: 2022 update. Ann Rheum Dis. 2023;82(1):3–18. 36357155 10.1136/ard-2022-223356

[31] Hashimoto M, Furu M, Yamamoto W, Fujimura T, Hara R, Katayama M, et al. Factors associated with the achievement of biological disease-modifying antirheumatic drug-free remission in rheumatoid arthritis: the ANSWER cohort study. Arthritis Res Ther. 2018;20(1):165. 30075810 10.1186/s13075-018-1673-1 PMC6091083

[32] Mori S, Okada A, Koga T, Ueki Y. Long-term outcomes after discontinuing biological drugs and Tofacitinib in patients with rheumatoid arthritis: A prospective cohort study. PLoS ONE. 2022;17(6):e0270391. 35737642 10.1371/journal.pone.0270391 PMC9223309

[33] Svensson B, Boonen A, Albertsson K, van der Heijde D, Keller C, Hafström I. Low-dose prednisolone in addition to the initial disease-modifying antirheumatic drug in patients with early active rheumatoid arthritis reduces joint destruction and increases the remission rate: a two-year randomized trial. Arthritis Rheum. 2005;52(11):3360–70. 16255010 10.1002/art.21298

[34] Montecucco C, Todoerti M, Sakellariou G, Scirè CA, Caporali R. Low-dose oral prednisone improves clinical and ultrasonographic remission rates in early rheumatoid arthritis: results of a 12-month open-label randomised study. Arthritis Res Ther. 2012;14(3):R112. 22584017 10.1186/ar3838 PMC3446489

[35] Asai S, Fujibayashi T, Oguchi T, Hanabayashi M, Hayashi M, Matsubara H, et al. Predictors of biologic discontinuation due to insufficient response in patients with rheumatoid arthritis who achieved clinical remission with biologic treatment: A multicenter observational cohort study. Mod Rheumatol. 2018;28(2):221–6. 28701065 10.1080/14397595.2017.1332558

[36] Visser K, Katchamart W, Loza E, Martinez-Lopez JA, Salliot C, Trudeau J, et al. Multinational evidence-based recommendations for the use of methotrexate in rheumatic disorders with a focus on rheumatoid arthritis: integrating systematic literature research and expert opinion of a broad international panel of rheumatologists in the 3E initiative. Ann Rheum Dis. 2009;68(7):1086–93. 19033291 10.1136/ard.2008.094474 PMC2689523

[37] Burmester GR, Kivitz AJ, Kupper H, Arulmani U, Florentinus S, Goss SL, et al. Efficacy and safety of ascending methotrexate dose in combination with adalimumab: the randomised CONCERTO trial. Ann Rheum Dis. 2015;74:1037–44. 24550168 10.1136/annrheumdis-2013-204769 PMC4431334

[38] Tamai H, Ikeda K, Miyamoto T, Taguchi H, Kuo CF, Shin K, et al. Reduced versus maximum tolerated methotrexate dose concomitant with adalimumab in patients with rheumatoid arthritis (MIRACLE): a randomised, open-label, non-inferiority trial. Lancet Rheumatol. 2023;5(4):e215–24. 38251524 10.1016/S2665-9913(23)00070-X

[39] Wolfe F, Kong SX, Watson DJ. Gastrointestinal symptoms and health related quality of life in patients with arthritis. J Rheumatol. 2000;27(6):1373–8. 10852256

