# Supplementary material for: Discontinuation vs. continuation of concomitant methotrexate in patients with rheumatoid arthritis on certolizumab pegol: results from a randomised, controlled trial
Source: Arthritis Res Ther. 2025 Apr 5;27:82. doi: 10.1186/s13075-025-03548-1 (PMC11972473; doi:10.1186/s13075-025-03548-1)
Supplement: Supplementary file 1 — Supplementary Material 1 [file 13075_2025_3548_MOESM1_ESM.docx]

**Supplemental Material**

**Discontinuation vs. Continuation of Concomitant Methotrexate in Patients with Rheumatoid Arthritis on Certolizumab Pegol: Results from a Randomised, Controlled Trial**

**Supplementary Material 1**

Estimated means (95% confidence interval [CI]) and mean (95% CI) changes from baseline (Δ) for clinical parameters.

|  | **CZP + MTX**  **(n = 41)** | **CZP**  **(n = 43)** | **P value** |
| --- | --- | --- | --- |
| **CDAI** |  |  |  |
| Week 0 | 2.9 (1.9 to 3.9) | 2.3 (1.3 to 3.2) | – |
| Week 12 | 3.0 (2.0 to 3.9) | 2.0 (1.0 to 2.9) | 0.935 |
| Week 24 | 2.7 (1.8 to 3.7) | 3.6 (2.7 to 4.5) | 0.153 |
| Week 36 | 2.7 (1.7 to 3.6) | 2.4 (1.5 to 3.4) | 0.997 |
| **ΔCDAI** |  |  |  |
| Week 12 | 0.1 (-0.7 to 0.9) | -0.3 (-1.1 to 0.4) | 0.935 |
| Week 24 | -0.1 (-0.9 to 0.7) | 1.3 (0.6 to 2.1) | 0.153 |
| Week 36 | -0.2 (-1.0 to 0.6) | 0.2 (-0.6 to 0.9) | 0.997 |
| **SDAI** |  |  |  |
| Week 0 | 3.0 (2.0 to 3.9) | 2.5 (1.5 to 3.4) | – |
| Week 12 | 3.0 (2.1 to 4.0) | 2.1 (1.1 to 3.0) | 0.900 |
| Week 24 | 2.8 (1.8 to 3.8) | 3.7 (2.8 to 4.7) | 0.214 |
| Week 36 | 2.7 (1.7 to 3.7) | 2.5 (1.6 to 3.5) | 1.000 |
| **ΔSDAI** |  |  |  |
| Week 12 | 0.1 (-0.7 to 0.9) | -0.4 (-1.2 to 0.4) | 0.900 |
| Week 24 | -0.1 (-0.9 to 0.7) | 1.3 (0.5 to 2.0) | 0.214 |
| Week 36 | -0.2 (-1.0 to 0.7) | 0.1 (-0.7 to 0.8) | 1.000 |
| **DAS28-CRP** |  |  |  |
| Week 0 | 1.6 (1.5 to 1.8) | 1.5 (1.4 to 1.7) | – |
| Week 12 | 1.6 (1.4 to 1.8) | 1.5 (1.3 to 1.7) | 1.000 |
| Week 24 | 1.6 (1.4 to 1.8) | 1.8 (1.6 to 1.9) | 0.143 |
| Week 36 | 1.6 (1.4 to 1.8) | 1.6 (1.4 to 1.8) | 0.941 |
| **ΔDAS28-CRP** |  |  |  |
| Week 12 | -0.0 (-0.2 to 0.1) | -0.1 (-0.2 to 0.1) | 1.000 |
| Week 24 | -0.0 (-0.2 to 0.1) | 0.2 (0.1 to 0.4) | 0.143 |
| Week 36 | -0.0 (-0.2 to 0.1) | 0.1 (-0.1 to 0.2) | 0.941 |

**Supplementary Material 1 continued**

|  | **CZP + MTX**  **(n = 41)** | **CZP**  **(n = 43)** | **P value** |
| --- | --- | --- | --- |
| **CRP, mg/dL** |  |  |  |
| Week 0 | 0.07 (-0.04 to 0.17) | 0.20 (0.10 to 0.31) | – |
| Week 12 | 0.06 (-0.05 to 0.17) | 0.12 (0.01 to 0.22) | 0.841 |
| Week 24 | 0.05 (-0.05 to 0.16) | 0.14 (0.04 to 0.24) | 0.372 |
| Week 36 | 0.06 (-0.05 to 0.17) | 0.10 (0.00 to 0.21) | 0.929 |
| **ΔCRP, mg/dL** |  |  |  |
| Week 12 | -0.01 (-0.20 to 0.18) | -0.09 (-0.27 to 0.09) | 0.841 |
| Week 24 | -0.02 (-0.21 to 0.17) | -0.06 (-0.24 to 0.12) | 0.372 |
| Week 36 | -0.01 (-0.20 to 0.18) | -0.10 (-0.28 to 0.08) | 0.929 |
| **MMP-3, ng/mL** |  |  |  |
| Week 0 | 50.1 (40.1 to 60.1) | 47.8 (38.2 to 57.5) | – |
| Week 12 | 51.3 (41.4 to 61.1) | 49.1 (39.6 to 58.6) | 1.000 |
| Week 24 | 50.0 (40.1 to 59.9) | 53.1 (43.7 to 62.6) | 0.921 |
| Week 36 | 49.9 (40.0 to 59.7) | 51.7 (42.2 to 61.1) | 0.900 |
| **ΔMMP-3, ng/mL** |  |  |  |
| Week 12 | 0.7 (-5.1 to 6.6) | 1.0 (-4.4 to 6.5) | 1.000 |
| Week 24 | 0.4 (-5.4 to 6.2) | 4.7 (-0.6 to 10.1) | 0.921 |
| Week 36 | -0.2 (-6.0 to 5.6) | 4.4 (-1.0 to 9.8) | 0.900 |

**Supplementary Material 1 continued**

|  | **CZP + MTX**  **(n = 41)** | **CZP**  **(n = 43)** | **P value** |
| --- | --- | --- | --- |
| **HAQ-DI** |  |  |  |
| Week 0 | 0.3 (0.1 to 0.4) | 0.3 (0.1 to 0.4) | – |
| Week 12 | 0.3 (0.1 to 0.4) | 0.2 (0.1 to 0.3) | 0.855 |
| Week 24 | 0.3 (0.1 to 0.4) | 0.3 (0.2 to 0.5) | 0.985 |
| Week 36 | 0.2 (0.1 to 0.4) | 0.2 (0.1 to 0.4) | 1.000 |
| **ΔHAQ-DI** |  |  |  |
| Week 12 | -0.0 (-0.1 to 0.1) | -0.1 (-0.2 to 0.0) | 0.855 |
| Week 24 | 0.0 (-0.1 to 0.1) | 0.0 (-0.1 to 0.1) | 0.985 |
| Week 36 | -0.1 (-0.2 to 0.0) | -0.1 (-0.2 to 0.0) | 1.000 |
| **EQ-5D** |  |  |  |
| Week 0 | 0.860 (0.813 to 0.907) | 0.860 (0.815 to 0.904) | – |
| Week 12 | 0.899 (0.852 to 0.947) | 0.897 (0.851 to 0.942) | 1.000 |
| Week 24 | 0.900 (0.853 to 0.948) | 0.868 (0.822 to 0.914) | 0.872 |
| Week 36 | 0.906 (0.859 to 0.953) | 0.895 (0.850 to 0.941) | 0.997 |
| **ΔEQ-5D** |  |  |  |
| Week 12 | 0.039 (-0.008 to 0.086) | 0.035 (-0.008 to 0.079) | 1.000 |
| Week 24 | 0.039 (-0.008 to 0.085) | 0.010 (-0.034 to 0.054) | 0.872 |
| Week 36 | 0.047 (-0.000 to 0.093) | 0.034 (-0.010 to 0.078) | 0.997 |

CZP: certolizumab pegol; MTX: methotrexate; CDAI: Clinical Disease Activity Index; SDAI: Simple Disease Activity Score; DAS28-CRP: Disease Activity Score with 28 joint counts with C-reactive protein; CRP: C-reactive protein; MMP-3: matrix metalloproteinase-3; HAQ-DI: Health Assessment Questionnaire Disability Index; EQ-5D: EuroQol-5 dimension.

**Supplementary Material 2**

Changes in Clinical Disease Activity Index (CDAI) score in patients who received rescue treatment due to CDAI score >10 in the CZP (i.e., methotrexate [MTX] discontinuation) group.

*Restarting MTX and intra-articular injection. †Restarting MTX.


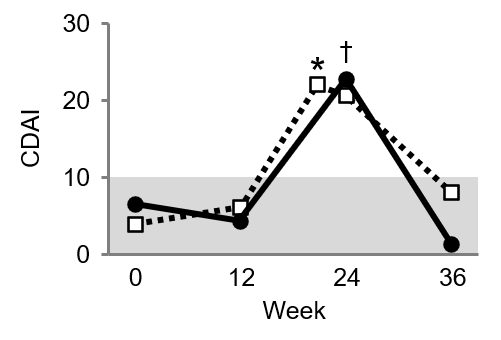


**Supplementary Material 3**

Estimated means (95% confidence interval [CI]) and mean (95% CI) changes from baseline (Δ) for Frequency Scale for Symptoms of Gastroesophageal reflux disease (FSSG) score and proportion of patients with FSSG score ≥8.

|  | **CZP + MTX**  **(n = 39)** | **CZP**  **(n = 43)** | **P value** |
| --- | --- | --- | --- |
| **FSSG score** |  |  |  |
| Week 0 | 4.2 (3.0 to 5.5) | 3.6 (2.4 to 4.7) | – |
| Week 12 | 3.0 (1.8 to 4.3) | 3.3 (2.1 to 4.5) | 0.930 |
| Week 24 | 3.2 (2.0 to 4.5) | 3.2 (2.0 to 4.4) | 0.992 |
| Week 36 | 3.0 (1.7 to 4.2) | 2.5 (1.3 to 3.6) | 1.000 |
| **ΔFSSG score** |  |  |  |
| Week 12 | -1.2 (-2.3 to -0.2) | -0.3 (-1.3 to 0.8) | 0.930 |
| Week 24 | -1.0 (-2.1 to 0.1) | -0.3 (-1.3 to 0.7) | 0.992 |
| Week 36 | -1.2 (-2.3 to -0.2) | -1.1 (-2.1 to -0.1) | 1.000 |
| **FSSG score ≥8, no (%)** |  |  |  |
| Week 0 | 10 (25.6) | 6 (14.0) | 0.182 |
| Week 12 | 4 (10.3) | 6 (15.0) | 0.526 |
| Week 24 | 5 (13.2) | 5 (12.2) | 0.898 |
| Week 36 | 6 (15.8) | 1 (2.4) | 0.034 |

CZP: certolizumab pegol; MTX: methotrexate.
